# Sibship assignment to the founders of a Bangladeshi *Catla catla* breeding population

**DOI:** 10.1186/s12711-019-0454-x

**Published:** 2019-04-29

**Authors:** Matthew G. Hamilton, Wagdy Mekkawy, John A. H. Benzie

**Affiliations:** 1 WorldFish, Jalan Batu Maung, 11960 Bayan Lepas, Penang Malaysia; 2 Animal Production Department, Faculty of Agriculture, Ain Shams University, Hadaeq Shubra, Cairo, 11241 Egypt; 3 School of Biological Earth and Environmental Sciences, University College Cork, Cork, Ireland

## Abstract

*Catla catla* (Hamilton) fertilised spawn was collected from the Halda, Jamuna and Padma rivers in Bangladesh from which approximately 900 individuals were retained as ‘candidate founders’ of a breeding population. These fish were fin-clipped and genotyped using the DArTseq platform to obtain, 3048 single nucleotide polymorphisms (SNPs) and 4726 silicoDArT markers. Using SNP data, individuals that shared no putative parents were identified using the program COLONY, i.e. 140, 47 and 23 from the Halda, Jamuna and Padma rivers, respectively. Allele frequencies from these individuals were considered as representative of those of the river populations, and genomic relationship matrices were generated. Then, half-sibling and full-sibling relationships between individuals were assigned manually based on the genomic relationship matrices. Many putative half-sibling and full-sibling relationships were found between individuals from the Halda and Jamuna rivers, which suggests that catla sampled from rivers as spawn are not necessarily representative of river populations. This has implications for the interpretation of past population genetics studies, the sampling strategies to be adopted in future studies and the management of broodstock sourced as river spawn in commercial hatcheries. Using data from individuals that shared no putative parents, overall multi-locus pairwise estimates of Wright’s fixation index (F_ST_) were low (≤ 0.013) and the optimum number of clusters using unsupervised K-means clustering was equal to 1, which indicates little genetic divergence among the SNPs included in our study within and among river populations.

**Electronic supplementary material:**

The online version of this article (10.1186/s12711-019-0454-x) contains supplementary material, which is available to authorized users.

## Background

In terms of quantity produced, *Catla catla* (Hamilton) is the sixth most important finfish aquaculture species, with approximately 2.8 × 10^6^ tons produced globally in 2015 [1]. It is primarily grown in South Asia, often on a small scale in polyculture with other species [2–4]. In spite of its economic importance, in a number of countries, including Bangladesh, the quality of catla seed produced in hatcheries has historically suffered from high levels of inbreeding, uncontrolled interspecific hybridisation and negative selection [2, 5]. In an effort to address these issues, in 2012, fertilised spawn was collected from the Halda, Jamuna and Padma (Ganges) rivers, as part of a project funded by the United States Agency for International Development (USAID) to restock Bangladeshi catla hatcheries with genetically diverse and non-inbred broodstock [6]. The collection of these fish was subsequently recognised as an opportunity to establish a breeding population for the long-term genetic improvement of the species. The aims of this study were to (1) develop and describe a panel of catla DArTseq-based silicoDArT and single nucleotide polymorphism (SNP) markers; (2) determine the extent of genetic relationships between, and assign putative sibship to, ‘candidate founders’ of the breeding population; and (3) examine molecular genetic variability among and within catla sampled from the three river systems.

## Methods

Fertilised spawn were collected in 2012 from the Halda, Jamuna and Padma rivers as part of a program to replace inbred broodstock in Bangladeshi hatcheries [6]. Fish from each river were reared separately by two commercial hatcheries in the case of the Halda and Padma, and by one hatchery in the case of the Jamuna. At 1 year of age, approximately three hundred catla individuals were randomly selected from each river as candidate founders of a breeding population. These fish were fin-clipped and samples were archived in 2015, as part of the routine husbandry of the breeding population. Fin-clips were obtained from fish that were anesthetized with clove oil by removing an approximately 2-mm-wide sample from the extremities of the dorsal fin. Subsequently, fish were placed in tanks for monitoring and released back into ponds once they had satisfactorily recovered from anaesthesia. All fish in the breeding population are managed in accordance with the Guiding Principles of the Animal Care, Welfare and Ethics Policy of the WorldFish Center [7].

For the purpose of the current study, in 2016, archived fin-clip samples were genotyped. Genotyping was conducted using the DArTseq platform [8] according to the laboratory procedures and analytical pipelines outlined in Lind et al. [9], except that the complexity reduction method involved a combination of PstI and SphI enzymes (SphI replacing HpaII used in Lind et al. [9]). Raw DArTseq data are available at 10.7910/DVN/6LEU9O [10].

Quality control procedures were implemented to ensure that only high-quality and informative SNPs, in approximate linkage equilibrium, were retained for analysis. First, SNPs with an observed minor allele frequency (MAF) lower than 0.05 or a rate of missing observations higher than 0.05 were excluded. Second, only one randomly-selected SNP was retained from each unique DNA fragment. Third, as a measure of linkage disequilibrium (LD), pairwise squared Pearson’s correlations (r^2^) of genotypic allele counts were computed, and then a random SNP from the pair with the highest r^2^ was excluded iteratively until all pairwise r^2^ values were lower than 0.2. Finally, SNPs that deviated from Hardy–Weinberg equilibrium (HWE) were filtered out. To achieve this, a preliminary analysis (method outlined below) was undertaken to identify and remove close relatives, and thus reduce the risk of false identification of SNPs with genotyping problems (see [11]). Then, data were converted to a ‘genind’ object using the ‘df2genind’ function (‘adegenet’ package, version 2.1.1 [12]) and the deviation from HWE for each SNP and sampled population was tested using the ‘hw.test’ function (‘pegas’ package, version 0.10 [13]). All the SNPs that significantly deviated from HWE in any sampled population were excluded (classical χ^2^ test; P < 0.05 after Dunn–Šidák correction).

To construct genomic relationship matrices (**G**), the method of VanRaden [14] was implemented using the code from Gondro [15] that was modified to replace missing observations in SNP data with the average of the observed allele frequency. The **G** matrix was constructed separately for individuals from each river. Clustering of genomic relationships using the ‘Ward2’ algorithm was implemented using the ‘hclust’ function [16]. Individuals were reordered according to clustering and heatmaps of genomic relationships between individuals were generated. These heatmaps revealed the presence of full-sibling and half-sibling relationships between the sampled individuals from each of the three river populations. To account for sibship in subsequent analyses and produce a pedigree for genetic analyses of the breeding population, sibship was assigned to individuals. Sibship was initially assigned using the program COLONY (version 2.0.6.4 [17]). For the COLONY analyses: (1) only SNPs with a MAF higher than 0.2 were retained, i.e. 571 from Halda, 569 from Jamuna, 518 from Padma; (2) individuals from different rivers were assumed to be unrelated; and (3) SNPs were assumed to be on separate chromosomes (i.e. unlinked). COLONY inputs were generated by means of the ‘write_colony’ function (‘radiator’ package version 0.0.11 [18]), using the default settings except that ‘update allele frequency’ was set to true [19]. Errors noted in the COLONY inputs generated by ‘write_colony’ were manually corrected.

Comparison of the **G** matrices with the pedigree-based additive (i.e. numerator) relationship matrices (**A**) [20], derived from COLONY sibship assignments, revealed that a large number of putatively full sibling relationships in the **G** matrices were assigned as half-siblings by COLONY, particularly in the case of the Padma river fish. This disparity was attributed to COLONY falsely splitting large full-sibship groups into multiple full-sibship groups [19, 21, 22]. However, by using the COLONY sibship assignments, putatively unrelated individuals were identified. For each river, these individuals were identified by (1) generating the **A** matrix (‘makeA’ function; ‘nadiv’ package version 2.16.0.0 [23]); (2) listing individuals that were unrelated (a_ij_ = 0) to other individuals in **A** and then removing these individuals from **A**; (3) appending to the list that was generated in step (2) the individual remaining in **A** with the lowest average relationship with the other individuals and then removing this individual and its relatives (a_ij_ > 0) from **A**; and (4) iteratively repeating step (3) until no individuals remained in **A**. Then, allele frequencies using data from the listed individuals only were taken as estimates of allele frequencies in the sampled river populations. These allele frequencies were provided as inputs to regenerate the **G** for each river. These **G** matrices were then used to manually assign sibship and dummy parents. This was achieved by (1) visually identifying groups of individuals with genomic relationships of approximately 0.5 and assigning these to full-sibling groups, and (2) visually identifying genomic relationships between these full-sibling groups of approximately 0.25 and defining these as half-siblings.

Data from putatively unrelated individuals only were used for the analysis of the genetic architecture of the river populations [11, 24]. Observed (H_obs_) and expected (H_exp_) heterozygosities were estimated by SNP and river of origin (‘summary’ function of ‘adegenet’). The significance of pairwise river of origin differences in mean H_exp_ were estimated (‘Hs.test’ function with n.sim = 999; ‘adegenet’ package) as was the difference between H_obs_ and H_exp_ within rivers (paired t-tests). Allelic richness and private allelic richness among rivers were compared visually using the rarefaction method implemented in ADZE [25].

After data conversion (‘tidy_genomic_data’ function; ‘radiator’ package), pairwise overall Wright’s [26] F_ST_ values between river populations [27], and bootstrap 95% confidence intervals derived from 2000 iterations, were computed (‘fst_WC84’ function default settings; ‘assigner’ package version 0.5.0 [28]). For the analysis of molecular variance (AMOVA), data were converted to genclone format with river of origin defined as the only stratum (‘as.genclone’ function; ‘poppr’ package version 2.7.1 [29]). Analysis of molecular variance was conducted using the ‘poppr.amova’ function (‘poppr’ package); implementing the default settings except that (1) variances within individuals were not calculated (i.e. within = FALSE), (2) the Hamming distance matrix was computed (i.e. dist = bitwise.dist(x)), and (3) the missing data threshold was set at 10% (i.e. cutoff = 0.1).

To investigate the possibility that a population structure other than that due to river of origin might fit the data better, unsupervised (K-means) clustering (‘find.clusters’ function; ‘adegenet’ package) was implemented using output from principal component analyses (PCA; ‘glPca’ function default settings with 500 principal components retained). In the implementation of the ‘find.clusters’ function, the maximum number of clusters was constrained to 20 and the number of randomly chosen starting centroids to be used in each run of the K-means algorithm was set at 1000. Then, the optimum number of clusters was identified as the level of K with the minimum Bayesian information criterion (BIC) [12].

## Results

In total, 3048 SNPs and 4726 silicoDArT markers were identified from 2630 and 4720 DArTseq-generated sequences (i.e. fragments), respectively (see Additional file 1: Table S1). Of the 3048 SNPs, 1347 SNPs remained after removal of the SNPs with more than 0.05 missing values and a MAF lower than 0.05, 1261 SNPs remained after removal of all but one SNP per fragment, 1034 SNPs remained after applying the constraint that all pairwise estimates of r^2^ ≤ 0.2, and ultimately 978 SNPs remained after removal of those that were not in putative HWE.

**Fig. 1:**
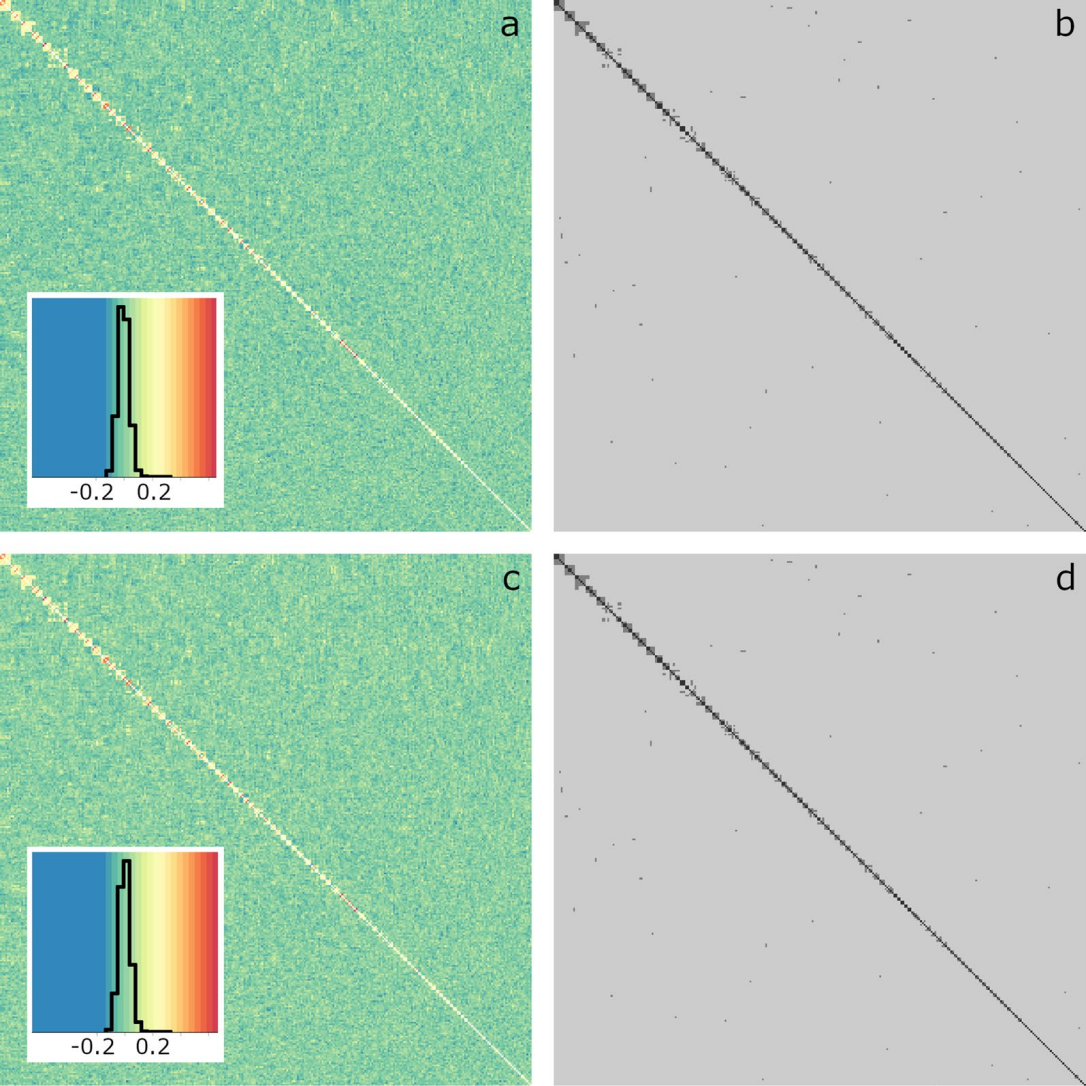
Heatmaps of relationship matrices for individuals from the Halda river. **a** Genomic relationship matrix (**G**) generated by using observed allele frequencies in all individuals, **b** COLONY-derived additive relationship matrix (**A**), **c G** using observed allele frequencies in individuals with no putative parents in common, and **d A** derived from manual sibship assignment. For **A** matrices, black represents a full sibling relationship (i.e. 0.50), dark grey represents a half sibling relationship (i.e. 0.25) and light grey represents no relationship (i.e. 0.00). Individuals in **a**–**d** are ordered according to clustering, using the ‘Ward2’ algorithm, of genomic relationships in **c**. Insets of **a** and **c** contain histograms of observed genomic relationships

**Fig. 2:**
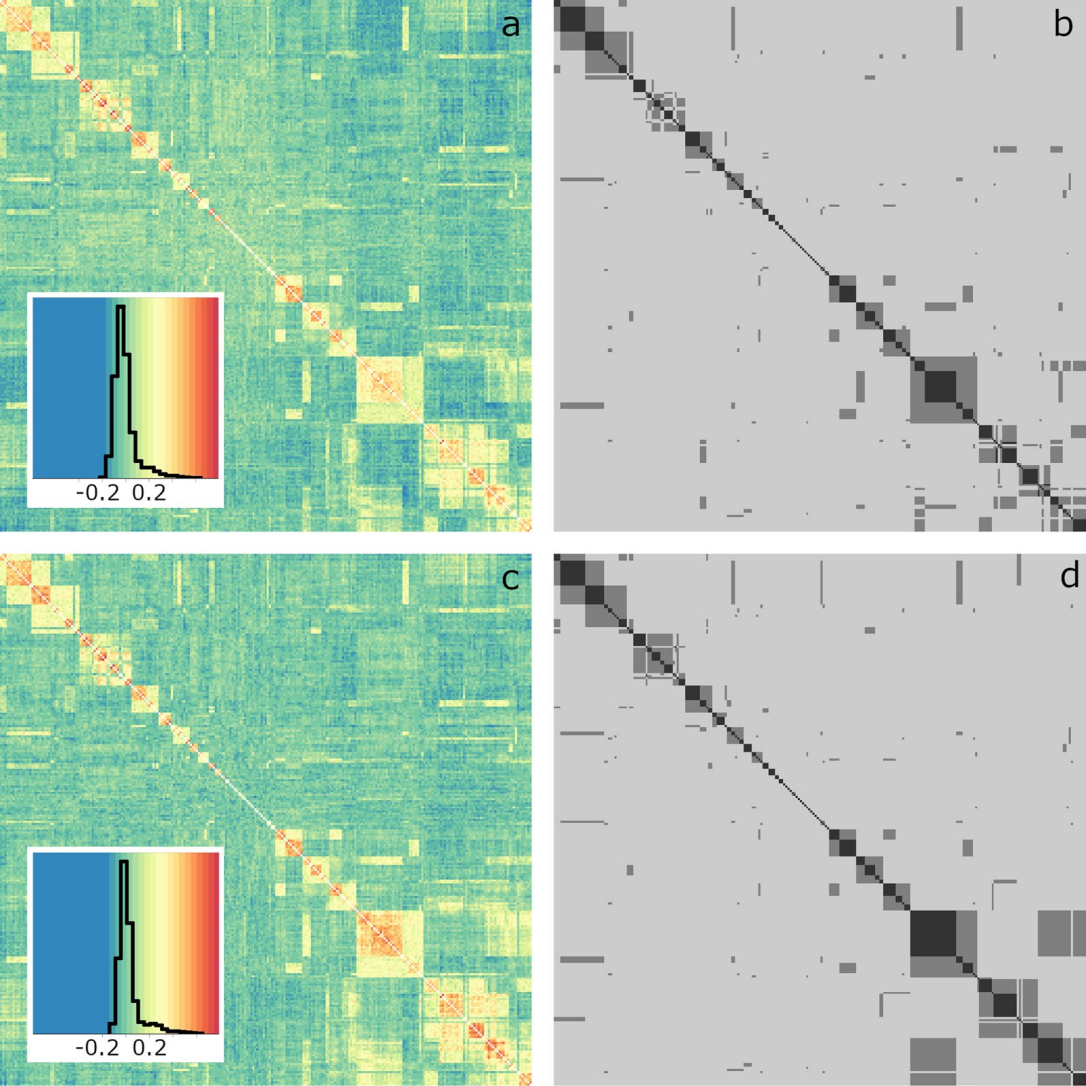
Heatmaps of relationship matrices for individuals from the Jamuna river. **a** Genomic relationship matrix (**G**) generated by using observed allele frequencies in all individuals, **b** COLONY-derived additive relationship matrix (**A**), **c G** using observed allele frequencies in individuals with no putative parents in common, and **d A** derived from manual sibship assignment. For **A** matrices, black represents a full sibling relationship (i.e. 0.50), dark grey represents a half sibling relationship (i.e. 0.25) and light grey represents no relationship (i.e. 0.00). Individuals in **a**–**d** are ordered according to clustering, using the ‘Ward2’ algorithm, of genomic relationships in **c**. Insets of **a** and **c** contain histograms of observed genomic relationships

**Fig. 3:**
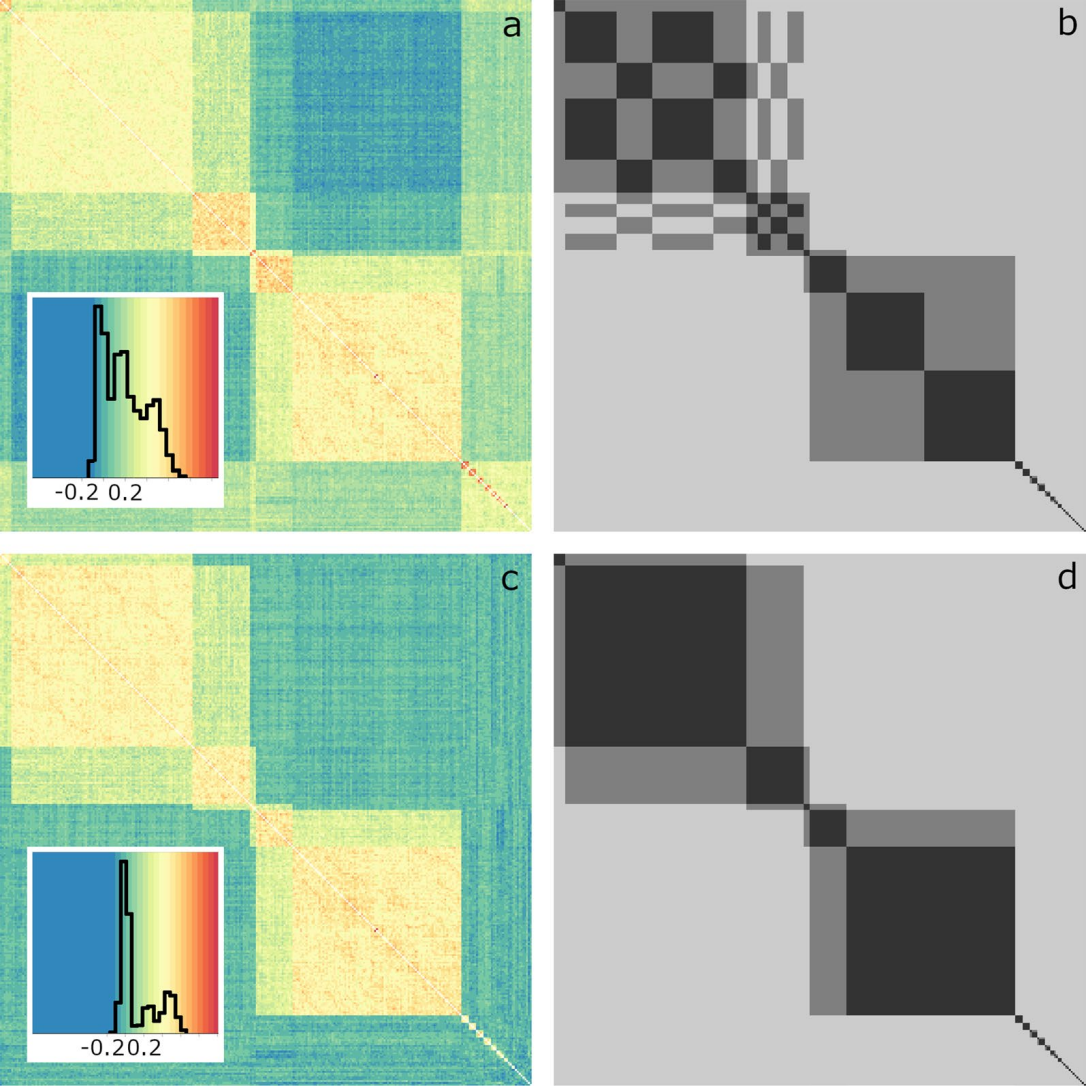
Heatmaps of relationship matrices for individuals from the Padma river. **a** Genomic relationship matrix (**G**) generated by using observed allele frequencies in all individuals, **b** COLONY-derived additive relationship matrix (**A**), **c G** using observed allele frequencies in individuals with no putative parents in common, and **d A** derived from manual sibship assignment. For **A** matrices, black represents a full sibling relationship (i.e. 0.50), dark grey represents a half sibling relationship (i.e. 0.25) and light grey represents no relationship (i.e. 0.00). Individuals in **a**–**d** are ordered according to clustering, using the ‘Ward2’ algorithm, of genomic relationships in **c**. Insets of **a** and **c** contain histograms of observed genomic relationships

Only 47 (18.4%) and 23 (8.0%) individuals with no putative parents in common were identified from the Jamuna and Padma fish, respectively, in contrast with 140 (46.8%) from the Halda. The **G** matrices that were generated using allele frequencies derived from individuals with no parents in common clarified the putative half- and full-sibling relationships between individuals (Figs. 1, 2 and 3), particularly in the case of the Padma river (Fig. 3). For this river, genomic relationships between individuals in small groups of closely-related individuals (i.e. putative full-sib groups) tended to be greater than those in large groups, when they were computed by using observed allele frequencies in all individuals (Fig. 3a). This trend was in accordance with the hypothesis that such estimates of allele frequencies in river populations were biased, due to the presence of large groups of closely-related individuals, and it was not found for genomic relationships computed by using observed allele frequencies in individuals with no putative parents in common (Fig. 3c). Notably, in the case of fish sourced from the Halda and Padma rivers, putative siblings were sourced from both hatcheries in which fish were reared, and thus replacement or inadvertent mixing of river-sourced fish with regular hatchery fish cannot explain the high degree of sibship observed.

More loci, for which the minor allele was absent prior to SNP quality control, were present in the dataset for Jamuna and Padma fish than in that for Halda fish (see Additional file 2: Figure S1); this is likely an artefact of the relatively small number of unrelated individuals sampled from these rivers. No significant differences (P > 0.082) between river populations in mean expected heterozygosities were detected, with values of 0.337 for the Halda, 0.337 for the Jamuna and 0.333 for the Padma river. Although significantly different from zero (P < 0.05), the difference between mean H_exp_ and H_obs_ was very small for all rivers (Halda mean H_obs_ = 0.324; Jamuna mean H_obs_ = 0.332; and Padma mean H_obs_ = 0.325). Furthermore, differences between river populations in allelic richness and private allelic richness were not substantive (see Additional file 1: Figure S2).

Analyses failed to reveal evidence of substantive genetic structure within or among the sampled populations. Analysis of molecular variance (AMOVA) indicated that variation among populations represented a very small (< 0.02) proportion of the total molecular marker variance, albeit significantly different from zero (P < 0.001). In addition, overall multi-locus pairwise estimates of Wright’s F_ST_ [26] were low [≤ 0.013; (see Additional file 1: Table S2)] and K-means clustering indicated the optimum number of clusters to be one (see Additional file 2: Figure S3).

## Discussion

The degree of sibship among Jamuna and Padma fish was higher than anticipated, and indicated that catla sampled from rivers as fertilised spawn are not necessarily representative of river populations. This has implications for the interpretation of past population genetics studies, the sampling strategies to be adopted in future studies, the management of broodstock sourced as river spawn in commercial hatcheries, and for pedigree-based genetic analyses and the management of inbreeding in the Bangladeshi catla breeding population. The high level of sibship observed among the Padma river individuals (Fig. 3) was particularly unexpected given that this is the largest of the three rivers sampled. However—due to the presence of a small number of spawning parents—a high degree of sibship might be expected, even in large river systems, if spawn is collected (1) at the tails of the spawning season, (2) from stretches of river in which the species is not common, or (3) from small river branches.

Poor performance of hatchery-produced seed in Bangladesh has been attributed, in part, to inbreeding caused by uncontrolled mating among a limited number of parents over multiple generations in closed hatchery populations [2, 5]. Our study indicates that this phenomenon may have been exacerbated by the presence of siblings in hatchery founder populations that have been sourced as spawn from rivers.

Sibship assignment and the generation of dummy parents were undertaken to improve pedigree-based analyses of breeding population data. These assignments were particularly problematic for the Jamuna river (Fig. 2), possibly due to (1) the mating of closely-related parents in the river or (2) the imprecise estimation of allele frequencies in the river population—due to the small number of individuals with no parents in common. In spite of this, the pedigree derived from our study is likely to represent a closer approximation of reality than the default assumption that individuals are unrelated. Encouragingly, we identified 210 founders with no parents in common, which represents a sizable base population for breeding purposes [30], in spite of a small number of putatively unrelated founders from two of the three rivers.

Pairwise inter-river F_ST_ estimates (< 0.013), using data from putatively unrelated individuals only, were generally lower than those previously published: Halda-Jamuna 0.014 [4], 0.014 [31], 0.017-0.034 [32] and 0.082 [33]; Halda-Padma 0.032 [4], 0.017 [31] and 0.052 [33]; and Jamuna-Padma 0.051 [4], 0.011 [31] and 0.054 [33]. In these past studies, samples were obtained as fertilised spawn or newly-hatched fry, and thus may contain unaccounted for sibship relationships and corresponding upward bias in F_ST_ estimates [11]. However, microsatellite or randomly amplified polymorphic DNA (RAPD) markers were used in these studies and thus comparisons with our SNP-derived estimates must be interpreted with caution, as SNPs often result in lower F_ST_ estimates than other markers [34].

The low molecular marker differentiation among rivers observed in our set of SNPs indicates a lack of genetic differentiation due to drift or adaptive selection and implies that there has been ongoing gene flow among the river systems. This was not unexpected in the case of the Padma and Jamuna rivers, since the Jamuna is a tributary of the Padma, but the Halda river is geographically and hydrologically isolated—although it is possible that natural gene flow between the Halda and other rivers has been exacerbated in recent history by translocation through restocking and aquaculture activities.

## Conclusions

In this study, (1) we have successfully developed and described a panel of catla DArTseq-based silicoDArT and single nucleotide polymorphism (SNP) markers for future genomic studies [10]; (2) we revealed that catla individuals collected as spawn from rivers cannot be assumed to be unrelated; (3) we assigned sibship and dummy parents to the ‘candidate founders’ of a breeding population; and (4) we found that molecular marker differentiation among sampled rivers was low. Our findings have been applied to modify the pedigree of a Bangladeshi catla breeding population to improve the accuracy of genetic parameter and breeding value estimates, and to minimise future inbreeding. Furthermore, the lack of genetic structure observed in our study, is likely to simplify any future implementation of genome-wide association studies (GWAS) and/or genomic selection [35].

## Additional files

**Additional file 1: Table S1.** Summary statistics for all genomic markers identified by DArTseq. Standard errors are in parentheses. **Table S2**: Overall multi-locus pairwise estimates of Wright’s F_ST_.

**Additional file 2:Figure S1.** Minor allele frequency across all river populations and within populations using (a) all SNPs and founders prior to quality control and (b) SNPs and founders used in population genetic analyses. The minor allele for each loci was identified in the dataset containing all rivers for (a) and (b) separately. White filled bars before zero represent SNPs for which the minor allele was absent (i.e. MAF is exactly 0). **Figure S2**. Mean number of (a) distinct alleles per locus and (b) private alleles per locus, as functions of standardized sample size for three rivers (excluding known relatives). **Figure S3**. Bayesian information criterion (BIC) against the number of clusters (K) from unsupervised K-means clustering.
