# Supplementary material for: Sibship assignment to the founders of a Bangladeshi Catla catla breeding population
Source: Genet Sel Evol. 2019 Apr 29;51:17. doi: 10.1186/s12711-019-0454-x (PMC6489195; doi:10.1186/s12711-019-0454-x)
Supplement: Supplementary file 1 — Additional file 1: Table S1. Summary statistics for all genomic markers identified by DArTseq. Standard errors are in parentheses. Table S2: Overall multi-locus pairwise estimates of Wright’s FST. [file 12711_2019_454_MOESM1_ESM.pdf]

Additional file 1: Peripheral Tables

Contents

Table S1. Summary statistics for all genomic markers identified by DArTseq. Standard errors are in parentheses.

Table S2. Overall multi-locus pairwise estimates of Wright's F_{ST}

Table S1. Summary statistics for all genomic markers identified by DArTseq. Standard errors are in parentheses.

	Marker Type	
	silicoDArT	SNP
Number of markers	4726	3048
Fragments sequenced containing one marker	4715	2305
Fragments sequenced with multiple markers	5	325
Unique fragments	4720	2630
Average fragment length (base pairs)	65.4 (0.17)	67.3 (0.16)
Fragment length minimum (base pairs)	20	20
Fragment length maximum (base pairs)	69	69
Polymorphic information content	0.28 (0.002)	0.24 (0.003)
Call rate	0.96 (0.001)	0.88 (0.003)
Reference read depth	25.5 (0.81)	21.7 (0.41)
SNP read depth	NA	14.1 (0.25)
Reproducibility*	0.998 (0.0001)	0.989 (0.0004)
Avg. missing data per individual (%)	4.39 (0.09)	12.4 (0.07)

* Reproducibility is the proportion of technical replicate assay pairs for which the marker score was consistent.

Table S2. Overall multi-locus pairwise estimates of Wright's F_{ST}

Population 1	Population 2	F_{ST} (95% confidence interval)
Halda	Jamuna	0.0131 (0.0113 - 0.0148)
Halda	Padma	0.0053 (0.0036 - 0.0072)
Jamuna	Padma	0.0017 (0.000 - 0.0032)
All		0.0096 (0.0082 - 0.0109)
