# Supplementary material for: Sibship assignment to the founders of a Bangladeshi Catla catla breeding population
Source: Genet Sel Evol. 2019 Apr 29;51:17. doi: 10.1186/s12711-019-0454-x (PMC6489195; doi:10.1186/s12711-019-0454-x)
Supplement: Supplementary file 2 — Additional file 2:Figure S1. Minor allele frequency across all river populations and within populations using (a) all SNPs and founders prior to quality control and (b) SNPs and founders used in population genetic analyses. The minor allele for each loci was identified in the dataset containing all rivers for (a) and (b) separately. White filled bars before zero represent SNPs for which the minor allele was absent (i.e. MAF is exactly 0). Figure S2. Mean number of (a) distinct alleles per locus and (b) private alleles per locus, as functions of standardized sample size for three rivers (excluding known relatives). Figure S3. Bayesian information criterion (BIC) against the number of clusters (K) from unsupervised K-means clustering. [file 12711_2019_454_MOESM2_ESM.pdf]

Additional file 2: Peripheral Figures

Contents

Figure S1 Minor allele frequency across all river populations and within popuations using a) all SNP and founders prior to quality control and b) SNP and founders used in popuation genetic analyses. The minor allele for each locus was identified in the dataset containing all rivers for a and b separately. White filled bars less than zero represent SNP loci in which the minor allele was absent (i.e. MAF is exactly zero).

Figure S2. The mean number of a) distinct alleles per locus and b) private alleles per locus, as functions of standardized sample size for three rivers (excluding known relatives).

Figure S3. Bayesian Information Criterion (BIC) against the number of clusters (K) from unsupervised K-means clustering.

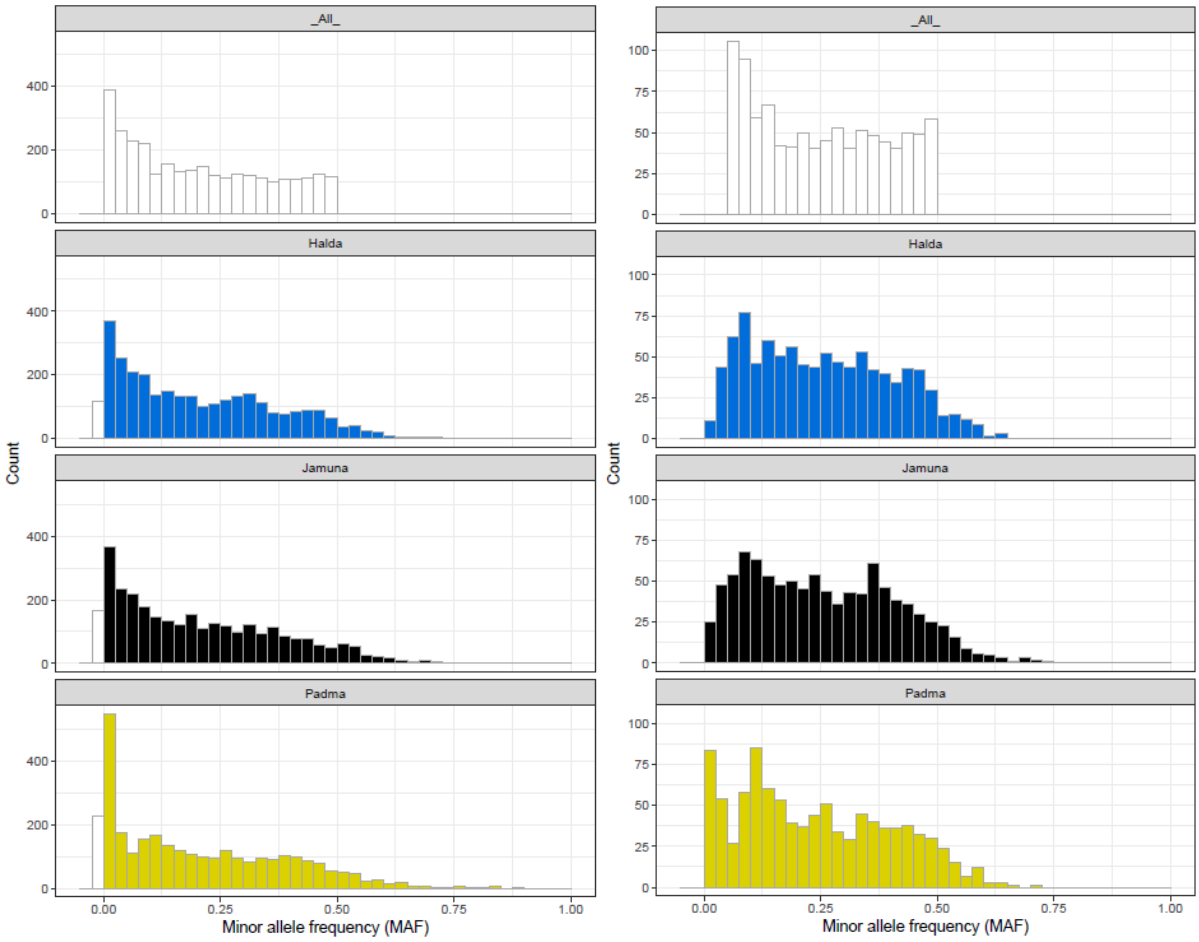

a)   b)

Figure S1 Minor allele frequency across all river populations and within popuations using a) all SNP and founders prior to quality control and b) SNP and founders used in popuation genetic analyses. The minor allele for each locus was identified in the dataset containing all rivers for a and b separately. White filled bars less than zero represent SNP loci in which the minor allele was absent (i.e. MAF is exactly zero).

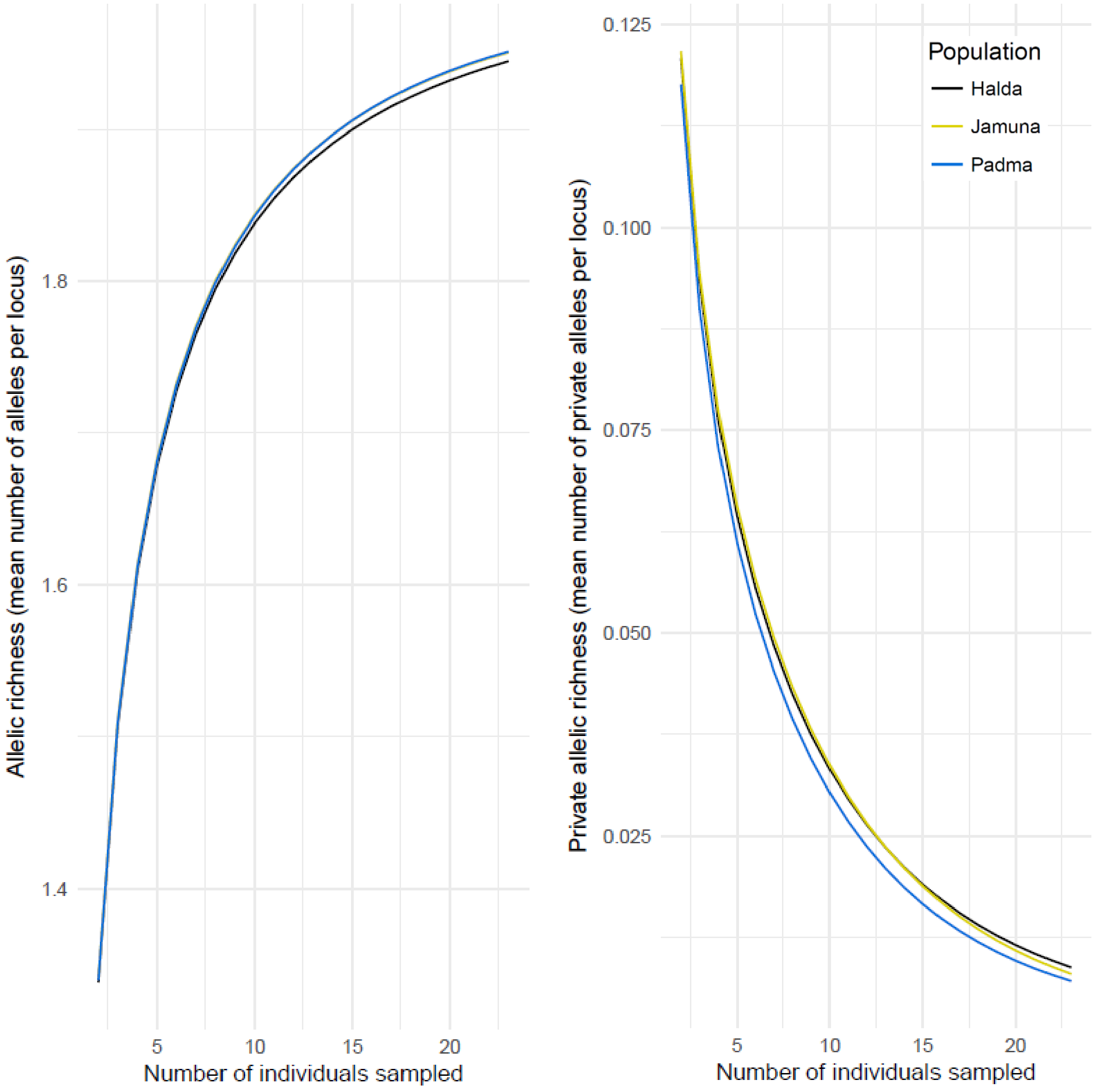

a) b)

Figure S2. The mean number of a) distinct alleles per locus and b) private alleles per locus, as functions of standardized sample size for three rivers (excluding known relatives).

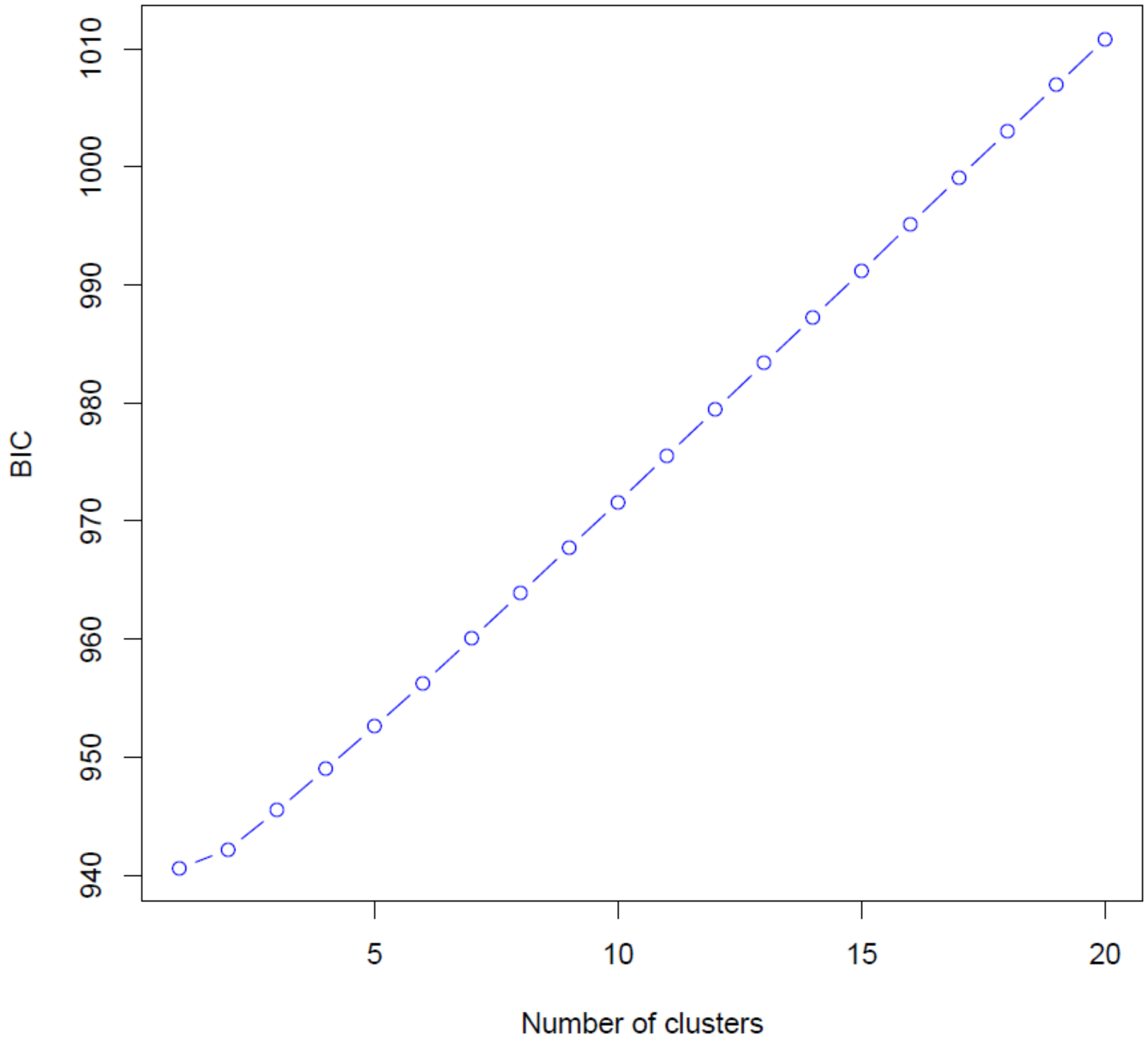

Figure S3. Bayesian Information Criterion (BIC) against the number of clusters (K) from unsupervised K-means clustering.
